# Healthcare utilization and psychiatric morbidity in violent offenders: findings from a prospective cohort study

**DOI:** 10.1007/s00127-022-02408-6

**Published:** 2022-12-27

**Authors:** André Tärnhäll, Jonas Björk, Märta Wallinius, Peik Gustafsson, Eva Billstedt, Björn Hofvander

**Affiliations:** 1 Lund Clinical Research on Externalizing and Developmental Psychopathology, Department of Clinical Sciences Lund, Lund University, Lund, Sweden; 2 Department of Forensic Psychiatry, Region Skåne, Malmö, Sweden; 3 Centre of Ethics, Law and Mental Health, Department of Psychiatry and Neurochemistry, University of Gothenburg, Gothenburg, Sweden; 4 Department of Occupational and Environmental Medicine, Lund University, Lund, Sweden; 5 Research Department, Regional Forensic Psychiatric Clinic, Växjö, Sweden; 6 Gillberg Neuropsychiatry Centre, Institute of Neuroscience and Physiology, University of Gothenburg, Gothenburg, Sweden; 7 Department of Clinical Sciences Lund, Lund University, Lund, Sweden

**Keywords:** Psychiatric morbidity, Violent offenders, Healthcare utilization, Psychotropic drugs, Criminal behavior

## Abstract

**Purpose:**

Although persistent offenders with histories of imprisonment and violence have disproportionate high rates of psychiatric disorders, little is known of their psychiatric healthcare utilization (HCU) and HCU-associated factors. This study aimed to explore psychiatric HCU, psychiatric morbidity, and psychotropic prescription drugs in violent offenders with a history of incarceration.

**Methods:**

Male offenders aged 18–25 (*n* = 266) imprisoned for violent and/or physical sexual offenses were clinically assessed in 2010–2012 and prospectively followed in Swedish national registries through 2017. Register-based information regarding HCU, psychiatric morbidity, and psychotropic drugs was tracked and compared with a general population group (*n* = 10,000) and across offending trajectory groups. Baseline risk factors were used to explain prospective psychiatric HCU in violent offenders.

**Results:**

Violent offenders used less general healthcare and psychiatric outpatient care, but more psychiatric inpatient care and were more often given psychiatric diagnoses and psychotropic drugs than the general population. Participants previously assigned to persisting offending trajectory groups had higher rates of psychiatric HCU than those assigned to a desisting trajectory. In multivariable regression models, psychiatric HCU was associated with anxiety disorders, prior psychiatric contact, placement in a foster home, psychopathic traits, low intellectual functioning, and persistent offending.

**Conclusions:**

Violent offenders are burdened by extensive and serious psychiatric morbidity and typically interact with psychiatric healthcare as inpatients rather than outpatients. Knowledge about their backgrounds, criminal behaviors, and psychiatric statuses can aid the planning of psychiatric services for this troublesome group.

**Supplementary Information:**

The online version contains supplementary material available at 10.1007/s00127-022-02408-6.

## Introduction

Despite prisoners’ suffering disproportionate levels of health problems [1], relatively little is known about their healthcare utilization (HCU) over time. Prison sentences offer healthcare staff a chance to meet this generally marginalized population and treat their old and new healthcare needs through prison services [2]. In prison, inmates appear to use both primary and specialist care more often than the general population [3–7]. Soon after their release, however, offenders are at particularly high risk of negative health outcomes, such as self-harm [8], drug overdose [9], and death [10]. HCU in recently released prisoners is elevated [5, 6, 11–14] and often associated with previous psychiatric conditions [15, 16]. Despite these findings during prisoners’ incarceration and soon after their release, knowledge of long-term HCU in people who have been in prison is scant.

Most psychiatric disorders are disproportionately prevalent in prisoners [17], decisively marking this as an area of high priority for research. Systematic reviews and meta-regressions in male prison samples have estimated prevalence rates of 3–4% for psychotic disorders, 9–12% for major depressive disorders, 24–38% for attention-deficit hyperactivity disorder (ADHD), 61–68% for personality disorders, and 22–38% for substance use disorders [17–20]. The World Health Organization has described mental health treatment during and after imprisonment as a pressing issue [2], yet psychiatric HCU is rarely reported. Kouyoumdjian and colleagues [6], however, showed that psychiatric inpatient care was markedly higher in prisoners than in the general population. Hassan and colleagues [21] also reported that the odds of being prescribed psychotropic drugs were over five times higher in male prisoners than in the general population. Psychiatric conditions, externalizing behaviors, and older age have all been associated with psychiatric HCU in prisoners [4, 7].

Not only are mental health problems an issue for offenders during incarceration, but offending is also associated with psychopathologies across the life-course including anxiety, depression, ADHD, psychosis, substance use, and personality disorders [22–25]. People recently released from prison are far more likely than the general population to be admitted to a psychiatric hospital [6]. Prospective information on outpatient psychiatric HCU in adults with a history of incarceration is scarce. Sodhi-Berry and colleagues [26] reported that around 16% of first-time adult offenders had been in contact with mental health services 5 years after sentencing and that prior contact with mental health services and substance use was associated with increased service interactions. Whether there are differences in psychiatric HCU determined by criminal trajectories is still an open question.

In a seminal article, Moffitt [27] proposed two paths of antisocial behaviors, one characterized by abnormal persistent levels of antisocial behaviors over the life-course, and the other seemingly limited to adolescence. She argued that those on the persistent path suffered far more developmental biopsychosocial risk factors and worse adult outcomes than individuals following the adolescent-limited path or individuals who never initiated an antisocial path. This taxonomy has received robust support in empirical studies [28], and it has been found that persistent offenders receive the most psychiatric healthcare, psychiatric diagnoses, and prescription drugs [24, 29–33].

Violent criminal behaviors can further complicate the developmental path in offenders [34] as they have been associated with decreased psychosocial functioning [35], increased risk of ill-health [36], and more HCU [37]. Violent offenders are often burdened by frequent criminal convictions and early life risk factors, and those who follow a persisting trajectory of criminal behaviors have more background risk factors than those who desist [38]. A Finnish birth cohort study showed that 30% of violent offenders had been treated at a psychiatric hospital before age 32, compared with 5% of non-offenders and 24% of non-violent offenders [39]. Still, few studies have explored psychiatric disorders and HCU in violent offender cohorts outside the forensic psychiatric context [40, 41], and fewer still have done so longitudinally. To our knowledge, no recent prospective prison cohort study has applied rich clinical psychiatric information to explore factors associated with longitudinal psychiatric HCU in offending trajectory groups, much less in violent offenders.

The general aim of this study was to prospectively explore psychiatric HCU, including psychiatric morbidity and prescriptions of psychotropic drugs, in a cohort of violent offenders with a history of incarceration in young adulthood. Specifically, we aimed to compare prospective HCU in a violent offender cohort (1) with the general population and (2) across offending trajectory groups. We further aimed (3) to analyze risk factors collected at baseline imprisonment that could prospectively predict psychiatric HCU in a violent offender cohort.

## Methods

### Participants

Two groups were included in this study, one cohort of male violent offenders (*n* = 266) and one general population comparison group (*n* = 10,000). The violent offender cohort was followed prospectively from their inclusion date (2010–2012) in the Development of Aggressive Antisocial Behavior Study (DAABS), a nationally representative Swedish longitudinal closed-cohort study of young adult violent offenders [40, 41]. At the time of inclusion, all were aged 18–25 and imprisoned for violent and/or ‘hands-on’ sexual offenses at a correctional facility in the western region of the Swedish Prison and Probation Service. During the period of baseline assessments, 420 individuals were imprisoned for such offenses in the catchment area: 109 declined participation and 42 were excluded because they need a Swedish interpreter (*n* = 23) or had an impending prison release (*n* = 19). Accurate personal identification numbers were not identified in three cases, making follow-up with those individuals impossible. The offender group thus consisted of 266 participants. Index offense, age, and basic demographic information did not differ between participants and non-participants. The comparison group was matched on age and gender and randomly drafted from the general population by Statistics Sweden. The comparison group was prospectively followed from 15 February 2010 (the date when the DAABS commenced). Both groups were followed prospectively through 2017. The average age at inclusion was 21.8 (SD = 1.9) in the DAABS cohort and 21.4 (SD = 2.0) in the comparison group. In the DAABS cohort and comparison group, 18 (7%) and 54 (0.5%) died, while 8 (3%) and 297 (3%) emigrated during the follow-up period, respectively.

### Procedure

Extensive baseline assessments of the DAABS participants, conducted by licensed clinical psychologists, included a full psychiatric diagnostic work-up following the LEAD (longitudinal, expert, all, data) principles [42]. Details of this data collection [40, 41, 43, 44] have been previously reported.

For the current study, we retrieved information on healthcare use from the National Patient Register (NPR) and the Prescribed Drug Register (PDR). The NPR includes information about all public and private specialist healthcare in Sweden, specialist inpatient care, and appointments to a medical doctor in specialist outpatient care, but no data on primary care. The PDR holds information about all prescribed drugs that have been dispensed from a Swedish pharmacy. The National Board of Health and Welfare oversees both the NPR and PDR.

Included baseline risk factors were gathered either at initial assessments or from retrospective register information (variable characteristics in Table S1).

### Measures

#### HCU

We retrieved prospective information on healthcare visits to outpatient and inpatient specialist care from the NPR, reported as count variables in different categories. When an outpatient care visit was followed by an inpatient visit in the same hospital, resulting in the same diagnoses on the same day, we counted only the inpatient visit.

#### Psychiatric morbidity

Psychiatric diagnoses, registered during specialist HCU, after baseline measures, were identified in the NPR based on ICD-10 codes (definitions in Table S2). Diagnoses were recorded as binary variables. All diagnoses from each healthcare visit were analyzed.

#### Prescribed drugs

Prescribed psychotropic drugs, dispensed after baseline measures, were identified through the PDR and categorized by the Anatomical Therapeutic Chemical classification system (definitions in Table S2). These binary variables represent any dispensation.

#### Follow-up time

The period between inclusion in the study to the end of 2017, death, or final emigration represented the follow-up time (person-time). Days in a psychiatric hospital were considered time-not-at-risk of further psychiatric HCU and subsequently subtracted from follow-up time. Information on death was gathered from the Cause of Death Register (National Board of Health and Welfare), emigration from the Migration Register (Statistics Sweden), and days in a psychiatric hospital from the NPR. In shaping this variable information, we conducted sensitivity analyses exploring the associations between length of prison sentence, death, migration, and number of psychiatric healthcare visits. Notably, no significant relationship was found between prospective psychiatric HCU and duration of the index prison sentence or total prior prison sentences, but a weak statistically significant positive relationship between prospective psychiatric HCU and duration of prison sentences after baseline was found (*ρ* = 0.21). Thus, long durations of imprisonment during the follow-up were not an obvious systematic barrier to psychiatric HCU. No relationship between prospective psychiatric HCU and early death, but a weak negative relationship between prospective psychiatric HCU and emigration (*ρ* =  − 0.13) was found in the DAABS cohort.

#### Psychiatric background

The Swedish translation of the Structured Clinical Interviews for DSM-IV-Axis I & II Disorders [45] was used to assess participants’ life-course psychiatric conditions at baseline interviews. Semi-structured interviews and file reviews were used to identify previous psychiatric HCU, including child, adolescent, and adult psychiatric HCU.

#### Socioeconomic background

Low education was defined as not having finished high school/upper secondary school. Never employed was defined as on no occasion having been registered as employed by any employer before baseline, with or without income. All employers in Sweden are obliged to annually register such information to Statistics Sweden. The variable was further adjusted with information on education above high school level, thus, if the participant had been in higher education they did not fill the criteria of never been employed. Information on employment and education was gathered from the Longitudinal Integration Database for Health Insurance and Labour Market Studies (Statistics Sweden). Information about country of birth was collected and reports as being born in or outside of Sweden.

#### Adversities and trauma

Information regarding placement in a foster home, institutional care, being bullied in school, parental substance abuse, being a witness or victim of physical parental violence, was collected during semi-structured interviews, self-reports, and file reviews.

#### Intellectual functioning

Wechsler Adult Intelligence Scale—Third Edition was used to assess intellectual functioning using the General Ability Index (GAI) score [46, 47]. The index has a standardized mean of 100 (SD = 15).

#### Conduct problems

We used the number of DSM-IV-TR [48] conduct disorder A symptoms at age 15 (range 0–15). Conduct disorder with symptom onset before age 10 was denoted *childhood-onset conduct disorder* in accordance with the DSM-IV-TR specifier [48] and coded into a binary variable. Information about bullying others (binary) and age at onset of alcohol and drug use (continuous) was gathered from interviews, self-reports, and file review.

#### Aggressive behaviors and psychopathic traits

Aggressive behaviors were assessed through the Life History of Aggression [49], a clinician rating scale with a total score range of 0–55. The Psychopathy Checklist-Revised (PCL-R) [50] was used to assess psychopathic traits (total score range = 0–40).

### Analytic strategy

For the first aim comparing violent offenders to the general population, descriptive data on HCU, psychiatric morbidity, and prescribed drugs were reported and analyzed with non-parametric tests (Pearson’s chi-square test and Mann–Whitney *U* rank-sum test). Effect sizes were reported (Cohen’s *d* [51] and odds ratios; OR) with a 95% confidence interval (CI). For the second specific aim, similar tests were used to analyze analogous data across trajectory groups based on total criminal activity in the DAABS cohort. The trajectory groups were identified in a previous report on the yearly rate of convicted crimes and offending trajectories of the DAABS cohort [38] using group-based trajectory modeling to approximate latent strata in individuals following similar trajectories [52]. That study found evidence of five offending trajectory groups:

Low-rate desisters (*n* = 83) characterized by relatively few convicted crimes and a desisting offending pattern:

Moderate-rate persisters (*n* = 91) with early incline in criminal behaviors, peaking at age 21, subsequent decline, and moderate rates of offending throughout the study period;

High-rate early-peak persisters (*n* = 36) with persistence in crime and high offending rates during the study period peaking at age 16;

High-rate late-peak persisters (*n* = 39) with generally high offending rates and a distinct peak of criminal activity at 21 years; and

High-rate inclining persisters (*n* = 17), the most criminally active group, with inclining yearly rates of criminal behavior during the study period.

To meet the third aim, we first analyzed risk factors that could help predict total psychiatric HCU in the DAABS cohort through univariable zero-inflated Poisson regression using information on follow-up time [53]. Each baseline risk factor’s association with the main outcome (i.e., total number of psychiatric healthcare visits), was analyzed separately and consecutively in the DAABS cohort. The main outcome was highly positively skewed (*p* < 0.001), with 64% of the DAABS participants unexposed (*n* = 171). As in any longitudinal research setting [54], the assumption in count regression models of observations being independent of one another was violated. To mitigate this, we used a robust, mixed-model extension estimator of variance [55] in all zero-inflated Poisson regression analyses, decreasing the risk of type I error. We report incident rate ratios (IRR) with 95% CIs and significance levels. After univariable analyses, Spearman’s pair-wise correlation was used to explore multicollinearity, and levels of tolerance for all baseline risk factors were considered for subsequent multivariable analyses. No multicollinearity was indicated. Subsequently, we conducted stepwise backward-selected multivariable zero-inflated Poisson regression analyses using risk factors passing the threshold of *p* < 0.3, excluding information for trajectory groups. The threshold for variables included in the final models was *p* < 0.1. We present two multivariable models, one excluding and one including information on offending trajectory group assignment. A *p* value of < 0.05 was considered statistically significant. Stata (version 15) was used in all analyses.

## Results

Prospective HCU, psychiatric diagnoses, and dispensed prescription drugs are reported in Table 1. The adjusted follow-up period was significantly longer in the comparison group (*M* = 7.74 years) than in the DAABS cohort (*M* = 6.18 years). With small effect sizes, the DAABS cohort was found to use less general and psychiatric outpatient healthcare than the comparison group. However, the DAABS cohort was found to use more psychiatric inpatient care than the comparison group, with a large effect size. Further analyses showed that 36% (*n* = 95) versus 13% (*n* = 1300) had used any psychiatric healthcare (OR = 3.71 [2.88–4.81]), while 30% (*n* = 80) versus 4% (*n* = 365) had been admitted to a psychiatric hospital (OR = 11.35 [8.57–15.04]). In an additional exploratory analysis of patterns of psychiatric healthcare, we found that only 7 (9%) of the 80 DAABS participants who had experienced psychiatric hospitalization had also received psychiatric outpatient care. In stark contrast, 329 (90%) of the 365 individuals in the comparison group who had received psychiatric inpatient care had also received psychiatric outpatient care.

**Table 1:** Prospective health care utilization, psychiatric morbidity, and prescribed drugs in violent offenders and a general population group

	DAABS (*n* = 266)	Comparison group (*n* = 10,000)	*p* value	Cohen’s *d* (95% CI)
Health care utilization	*M* (*SD*)	*M* (*SD*)		
Total health care visits	3.61 (7.3)	5.69 (9.6)	< 0.001*	− 0.22 (− 0.34 to − 0.09)
Psychiatric visits	1.55 (3.8)	1.13 (5.5)	< 0.001*	.08 (− 0.04 to 0.20)
Psychiatric outpatient visit	0.18 (0.9)	1.02 (4.9)	0.02*	− 0.17 (− 0.29 to − .05)
Psychiatric inpatient visits	1.37 (3.7)	0.11 (1.1)	< 0.001*	1.04 (0.92 to 1.17)

	*n* (%)	*n* (%)		OR (95% CI)
Psychiatric morbidity				
Any psychiatric disorder	99 (37)	1350 (14)	< 0.001*	3.80 (2.94–4.90)
Major depressive disorders	17 (6)	587 (6)	0.72	1.09 (0.67–1.79)
Anxiety disorders	29 (11)	589 (6)	0.001*	1.96 (1.32–2.89)
Severe stress reactions and adjustment disorders	20 (8)	219 (2)	< 0.001*	3.63 (2.27–5.82)
Post-traumatic stress disorder	3 (1)	29 (0.3)	0.02*	3.92 (1.26–12.20)
Bipolar disorders	4 (2)	68 (1)	0.11	2.23 (0.84–5.93)
Psychotic disorders	22 (8)	108 (1)	< 0.001*	8.26 (5.15–13.24)
Primary psychotic disorders	15 (6)	90 (1)	< 0.001*	6.58 (3.78–11.46)
Substance-induced psychotic disorders	14 (5)	39 (0.4)	< 0.001*	14.19 (7.68–26.24)
Alcohol use disorder	28 (11)	256 (3)	< 0.001*	4.48 (2.98–6.74)
Drug use disorders	72 (27)	320 (3)	< 0.001*	11.23 (8.39–15.03)
Personality disorders	14 (5)	179 (1)	< 0.001*	6.98 (3.93–12.40)
ADHD	33 (12)	348 (3)	< 0.001*	3.92 (2.69–5.73)
Autism	1 (.4)	182 (2)	0.08	0.20 (0–1.16)
Intellectual disability	2 (1)	64 (1)	0.82	1.18 (0–4.39)
Prescribed drugs				
Any psychotropic	178 (67)	4100 (41)	< 0.001*	2.91 (2.24–3.77)
Narcotics, classified	158 (59)	3372 (34)	< 0.001*	2.88 (2.24–3.68)
Analgesics	111 (42)	2566 (26)	< 0.001*	2.07 (1.62–2.66)
Antiepileptics	48 (18)	297 (3)	< 0.001*	7.19 (5.16–10.03)
Antipsychotics	50 (19)	338 (3)	< 0.001*	6.61 (4.78–9.16)
Anxiolytics	79 (30)	1212 (12)	< 0.001*	3.06 (2.34–4.01)
Antidepressants	81 (30)	1420 (14)	< 0.001*	2.65 (2.03–3.45)
Mood stabilizers	22 (8)	170 (2)	< 0.001*	5.21 (3.30–8.24)
Psychostimulants	46 (17)	334 (3)	< 0.001*	0.05 (4.33–8.46)
Drugs used in alcohol use disorder	14 (5)	77 (1)	< 0.001*	17.16 (4.03–12.73)
Drugs used in opioid use disorder	4 (2)	12 (0.1)	< 0.001*	12.71 (4.29–37.65)

*p < 0.05

*ADHD* attention-deficit hyperactivity disorder,* OR* odds ratio,* CI* confidence interval

Most psychiatric disorders were more often diagnosed in the DAABS cohort than in the comparison group. No diagnostic category was found to be more common in the comparison group. Notably, the DAABS cohort had much higher odds of receiving diagnoses of drug use disorders (27 vs. 3%, OR = 11.23 [8.39–15.03]), personality disorders (5 vs. 1%, OR = 6.98 [3.93–12.40]), or psychotic disorders (8 vs. 1%, OR = 8.26 [5.15–13.24]) than the comparison group. We also found that 6% in the DAABS cohort had received an opioid substance use diagnosis (defined as ICD-codes F11) versus 0.6% in the comparison group (OR = 9.21 [5.31–16.00]).

The DAABS cohort was also more likely than the comparison group to have had any psychotropic drug dispensed (OR = 2.91 [2.24–3.77]), drugs used in treatment of opioid (OR = 12.71 [4.29–37.65]) or alcohol use disorders (OR = 7.16 [4.03–12.73]), antiepileptics (OR = 7.19 [5.16–10.03]), antipsychotics (OR = 6.61 [4.78–9.16]), or psychostimulants (OR = 6.05 [4.33–8.46]).

Prospective HCU, psychiatric diagnoses, and dispensed prescription drugs across cohort trajectory groups are reported in Table 2. The adjusted follow-up period did not differ significantly across trajectory groups. DAABS participants assigned to the persisting trajectory groups used more healthcare, were more likely to have received a psychiatric diagnosis, and been dispensed psychotropic prescribed drugs than the desisting groups.

**Table 2:** Prospective health care utilization, psychiatric morbidity, and prescribed drugs across groups of violent offenders

Trajectory groups	L-D (*n* = 83)	M-P (*n* = 91)	H-LP (*n* = 39)	H-EP (*n* = 36)	I-P (*n* = 17)	*p* value
Health care utilization	*M* (*SD*)					
Total health care visits	1.27 (1.69)	4.22 (8.63)	5.36 (10.78)	2.50 (3.29)	10.18 (8.67)	< 0.001*
Psychiatric visits	0.27 (0.52)	1.92 (4.53)	2.10 (4.24)	1.20 (2.41)	5.35 (6.51)	< 0.001*
Outpatient psychiatric visits	0.07 (0.26)	0.22 (0.76)	00.03 (0.16)	0.19 (0.86)	0.88 (2.71)	0.35
Inpatient psychiatric visits	0.19 (0.48)	1.70 (4.50)	2.08 (4.25)	1.00 (2.34)	4.47 (6.09)	< 0.001*
Psychiatric morbidity	*n* (*%*)					
Any psychiatric disorder	14 (17)	40 (44)	17 (44)	15 (42)	13 (76)	< 0.001*
Major depressive disorders	3 (4)	8 (9)	2 (5)	2 (6)	2 (12)	0.57
Severe stress reactions and adjustment disorders	3 (4)	9 (10)	5 (12)	0 (0)	3 (18)	0.05*
Bipolar disorders	1 (1)	2 (2)	0 (0)	1 (3)	0 (0)	0.81
Psychotic disorders	1 (1)	7 (8)	4 (10)	3 (8)	7 (41)	< 0.001*
Alcohol use disorder	5 (6)	9 (10)	6 (15)	5 (14)	3 (18)	0.39
Drug use disorders	3 (4)	29 (32)	4 (36)	13 (36)	13 (76)	< .001*
Personality disorders	0 (0)	7 (8)	2 (5)	2 (6)	3 (18)	0.03*
ADHD	4 (5)	13 (14)	5 (13)	6 (17)	5 (29)	0.04*
Prescribed drugs						
Any psychotropic	47 (57)	60 (66)	29 (74)	27 (75)	15 (88)	0.05*
Narcotics, classified	40 (48)	57 (63)	24 (62)	22 (61)	15 (88)	0.03*
Analgesics	31 (37)	38 (42)	16 (41)	15 (42)	11 (65)	0.36
Antiepileptics	4 (5)	22 (24)	12 (31)	5 (14)	5 (29)	0.001*
Antipsychotics	9 (11)	20 (22)	5 (13)	8 (22)	8 (47)	0.01*
Anxiolytics	17 (20)	31 (34)	12 (31)	11 (31)	8 (47)	0.15
Antidepressants	10 (12)	36 (40)	13 (33)	15 (42)	7 (41)	< 0.001*
Mood stabilizers	3 (4)	11 (12)	5 (13)	2 (6)	1 (6)	0.23
Psychostimulants	10 (12)	16 (18)	5 (13)	6 (17)	9 (53)	0.002*
Drugs, alcohol use disorder	4 (5)	7 (8)	1 (3)	2 (6)	0 (0)	0.62
Drugs, opioid use disorder	0 (0)	2 (2)	1 (3)	0 (0)	1 (6)	0.33

*p < 0.05

*L-D* low-rate desisters, *M-P* moderate-rate persisters, *H-LP* high-rate late-peak persisters, *H-EP* high-rate early-peak persisters, *H-IP* high-rate inclining persisters, *ADHD* attention-deficit hyperactivity disorder

Univariable zero-inflated Poisson regression models studying the effect of single baseline risk factors at or before inclusion in the study on total prospective psychiatric healthcare visits in the DAABS cohort are presented in Table 3. We found an increased incidence of psychiatric HCU in participants with baseline anxiety disorders (IRR = 2.77 [1.10–7.00]) and mood disorders (IRR = 2.43 [1.08–5.49]) than in those without. The strongest association with this outcome was found with prior psychiatric HCU (IRR = 4.30 [1.42–12.97]). Furthermore, placement in a foster home (IRR = 2.69 [1.57–4.61]), never having been employed (IRR = 1.87 [1.03–3.38]), having experienced family-related physical violence in childhood (IRR = 1.80 [1.06–3.07]), and being bullied (IRR = 1.76 [1.01–3.08]) were significantly associated with psychiatric HCU, as were scores on the GAI (IRR = 0.97 [0.95–0.99]) and the PCL-R (IRR = 1.04 [1.01–1.08]). With each decrease in GAI score or with each additional PCL-R score the incidence of psychiatric HCU increased by 3 or 4%, respectively.

**Table 3:** Zero-inflated Poisson regression models using follow-up time to study the effect of trajectory groups and single risk factors from the DAABS baseline on total psychiatric health care visits in the DAABS cohort

	Effect size IRR (95% CI)	*p* value
Trajectory group		
Low-rate desisters	(reference group)	
Moderate-rate persisters	9.21 (5.12–16.59)	< 0.001*
High-rate late-peak persisters	8.41 (4.31–16.39)	< 0.001*
High-rate early-peak persisters	5.42 (2.79–10.51)	< 0.001*
High-rate inclining persisters	13.98 (7.84–24.91)	< 0.001*
Psychiatric background		
Mood disorders	2.43 (1.08–5.49)	0.03*
Anxiety disorders	2.77 (1.10–7.00)	0.03*
Psychotic disorders	1.28 (0.67–2.43)	0.45
Substance use disorders	3.07 (0.87–10.88)	0.08
Personality disorders	1.58 (0.87–2.87)	0.14
ADHD	1.75 (0.84–3.65)	0.14
Autism	1.51 (0.43–5.27)	0.52
Prior psychiatric care	4.30 (1.42–12.97)	0.01*
Socioeconomic background		
Low education	0.88 (0.44–1.77)	0.72
Never employed	1.87 (1.03–3.38)	0.04*
Born outside Sweden	1.04 (0.57–1.89)	0.90
Adversities and trauma		
Placement in a foster home	2.69 (1.57–4.61)	< 0.001*
Placement in institutional care	1.66 (0.91–3.05)	0.10
Being bullied	1.76 (1.01–3.08)	0.05
Parental substance abuse	1.17 (0.68–2.02)	0.57
Parental violence, witness	1.04 (0.60–1.80)	0.89
Parental violence, victim	1.80 (1.06–3.07)	0.03*
Intellectual functioning		
General ability index	0.97 (0.95–0.99)	0.001*
Conduct problems		
Childhood-onset conduct disorder	1.24 (0.72–2.14)	0.45
Number of conduct disorder symptoms	1.03 (0.95–1.12)	0.41
Age at onset alcohol use	0.94 (0.83–1.07)	0.37
Age at onset substance use	0.97 (0.80–1.17)	0.72
Bullied others	0.94 (0.54–1.65)	0.84
Aggressive behaviors and psychopathic traits		
Life history of aggression score	1.01 (0.98–1.04)	0.50
Psychopathy checklist–revised score	1.04 (1.01–1.08)	0.02*

*p < 0.05

*CI* confidence interval, *DAABS* Development of Aggressive Antisocial Behavior Study, *IRR* incidence rate ratio, *ADHD* attention-deficit hyperactivity disorder

For sensitivity and transparency purposes, we report supplementary univariable zero-inflated Poisson regression analyses of information collapsed into overarching categories and additional information on conduct problems in Table S3.

Multivariable zero-inflated Poisson regression models are presented in Table 4. Taking the backward-selected variables into account in model 1, the incidence of psychiatric HCU increased in the total group with baseline anxiety disorders (IRR = 2.36 [1.19–4.67]), prior psychiatric HCU (IRR = 2.07 [1.02–4.18]), placement in a foster home (IRR = 2.60 [1.53–4.42]), and each additional PCL-R score (IRR = 1.06 [1.02–1.10]), and decreased with each additional GAI score (IRR = 0.97 [0.95–0.99]). By introducing offending trajectory groups in model 2, in which *low-rate desisters* was used as the reference group, all associations except prior psychiatric HCU remained significant. Being assigned to any three of the four persistent trajectory groups increased the incidence of psychiatric HCU on a statistically significant level: *moderate-rate persisters* (IRR = 4.02 [2.03–7.97]), *high-rate late-peak persisters* (IRR = 2.73 [1.33–5.63]), and *high-rate inclining persisters* (IRR = 3.60 [1.34–9.67]). Elevated incidence of psychiatric HCU was also suggested for *high-rate early-peak persisters*: (IRR = 1.82 [0.79–4.18]), but this result was more statistically uncertain.

**Table 4:** Multivariable zero-inflated Poisson regression model using follow-up time to explain total psychiatric healthcare visits through risk factors and trajectory groups identified in the DAABS cohort

	Model 1 IRR (95% CI)	*p* value	Model 2 IRR (95% CI)	*p* value
Risk factors				
Anxiety disorders	2.36 (1.19–4.67)	0.01*	2.08 (1.18–3.67)	0.01*
Prior psychiatric care	2.07 (1.02–4.18)	0.04*	1.61 (0.85–3.05)	0.15
Placement in a foster home	2.60 (1.53–4.42)	< 0.001*	2.26 (1.36–3.75)	0.002*
General Ability Index	0.97 (0.95–0.99)	< 0.001*	0.97 (0.95–0.99)	0.002*
Psychopathy Checklist–Revised score	1.06 (1.02–1.10)	0.001*	1.06 (1.01–1.11)	0.01*
Trajectory groups				
Low-rate desisters			(reference group)	
Moderate-rate persisters			4.02 (2.03–7.97)	< 0.001*
High-rate late-peak persisters			2.73 (1.33–5.63)	0.007*
High-rate early-peak persisters			1.82 (0.79–4.18)	0.16
High-rate inclining persisters			3.60 (1.34–9.67)	0.01*

*p < 0.05

*CI* confidence interval, *DAABS* Development of Aggressive Antisocial Behavior Study, *IRR* incidence rate ratio

## Discussion

We present evidence that a violent offender cohort with a history of incarceration in young adulthood used less total healthcare, but more psychiatric healthcare, largely attributable to an excess of inpatient psychiatric HCU, than matched individuals from the general population. While merely one in twenty in the comparison group had any inpatient psychiatric HCU, one in three in the DAABS cohort had been admitted to a psychiatric hospital during approximately 6 years of follow-up (OR = 11.35 [8.57–15.04]) [39]. Despite the increased levels of psychiatric HCU in the DAABS cohort, they used far less outpatient psychiatric care than the comparison group [26]. This HCU pattern is further demonstrated by the almost total lack of outpatient care by DAABS cohort participants with experience of inpatient psychiatric care, as opposed to the balance in the two types of psychiatric care in the general population. The lower total HCU in the DAABS cohort than in the general population group does not likely represent lower healthcare needs in the offender cohort [1, 6, 33]. Rather, it may be a result of the numerous barriers to HCU reported in offender populations [56, 57].

The DAABS cohort was overrepresented in most psychiatric disorders and in all categories of prescribed psychotropic drugs compared with the control group. These results confirm the burden on this population of extensive and serious mental health issues [17, 21, 22]. Levels of lifetime prevalence of psychiatric disorders, other than psychotic disorders, were distinctly higher in a previous report regarding the DAABS cohort [41] than in the prospective register-based data presented here: drug use disorder (84 vs. 27%), personality disorder (67 vs. 5%), and anxiety disorders (52 vs. 11%). Considering the psychiatric backgrounds of those in the DAABS cohort [40, 41], the prospective level of psychiatric HCU, especially outpatient care, is low. The high levels of psychotropic drugs dispensed to this population, however, reflect a more pronounced psychiatric morbidity in the DAABS cohort than the data captured by specialist care diagnostic data. This could indicate a pattern in the DAABS cohort of using primary care as the dominant referral for mental health concerns, including serious mental illnesses. We also observed that 17% of this cohort were dispensed psychostimulants on at least one occasion, slightly higher than the 12% of the general population with a prospective diagnosis of ADHD, though much lower than the 43% of the DAABS cohort [40] displaying ADHD symptomatology in young adulthood. Here, it is worth noting that continuity in psychopharmacological treatment for ADHD with psychostimulants has been associated with a reduced risk of reoffending [58, 59]. Further, we note that violent offenders with substance use disorders are commonly prescribed narcotics-classified drugs, although they are rarely (2%) dispensed drugs used to treat substance use disorders from a pharmacy or diagnosed with opioid use disorders (6%) in a specialist care setting. Considering previous reports of heroin (34%) and other opioid (42%) use disorders in the DAABS cohort at baseline [41] and previous findings of a possible protective effect of opioid substitution treatments on violent reoffending [59], the number of individuals in such treatment seems surprisingly low.

As expected, persistent offenders used more specialist healthcare than other offenders [29, 33]. However, the low levels of total and psychiatric HCU and psychotropic medication in the desisting trajectory group were unanticipated, as it has been reported that desisting groups use healthcare at similar or slightly higher levels as non-offenders [29, 33]. This could reflect a particular reluctance to seek healthcare and/or lower healthcare needs in the desisting trajectory groups, but those questions are beyond the scope of this study. The lower HCU in the high-rate early-peak persisters trajectory group compared with the other persistent trajectory groups was also unexpected, as a previous study [38] found prominent levels of markers of social marginalization that typically increase the risk of ill-health in this trajectory group including low education, placements in institutional care, and early onset of various conduct problems.

Several baseline risk factors predicted psychiatric HCU univariably in the DAABS cohort, including those associated with psychiatric background, socioeconomic background, adversities and trauma, intellectual functioning, and psychopathic traits. Individuals suffering from psychotic disorders are often in need of psychiatric management; unexpectedly, baseline psychotic disorders in the DAABS cohort were not found to be associated with prospective psychiatric HCU. In the multivariable analyses, baseline anxiety disorders, prior psychiatric care, placement in a foster home, lower intellectual functioning, and increased levels of psychopathic traits increased the incidence of psychiatric HCU during the follow-up period. Prior psychiatric care utilization is a relatively established factor associated with HCU [4, 7, 26], and this study supports those findings. Anxiety disorders, more common in offender populations than non-offenders, and most common in persistent offenders [24], can be highly disabling and problematic in both post-release reintegration after prison and recovery from substance use disorders. Experiences of foster home and other out-of-home placements have been associated with a wide range of negative outcomes later in life, including psychiatric morbidity [60, 61]. As institutional placements have been associated with a greater risk of psychiatric morbidity than foster home placements [61], it is worth noting that in this study, only foster home placements, not institutional placements, were associated with psychiatric HCU.

Each additional point of GAI in this study was associated with a 3% decreased incidence of psychiatric HCU, so one additional standard deviation (15 pts) in the GAI meant a 45% decrease. This finding is similar to a previous study in the male general population [62], which reported that each point decrease in intellectual functioning in young adulthood increased the hazard ratio of subsequent psychiatric inpatient care. In contrast to Schrader and colleagues’ [63] finding that psychopathy was not associated with HCU during or after incarceration, we found that each additional point on the PCL-R (range 0–40) increased the incidence of prospective psychiatric HCU by 6%. Individuals with several psychopathic traits are more likely to suffer from psychiatric comorbidities, including co-existing substance use disorders, rendering a broad spectrum of psychiatric healthcare needs [64], which in part could aid in understanding their association with psychiatric HCU. Univariable analyses indicated an elevated incidence of psychiatric HCU in participants assigned to persistent as opposed to desisting trajectory groups. In the second multivariable model, in which the backward-selected variables in model 1 regarding psychiatric background were introduced, this association remained. This indicates that several paths of persistent criminal behaviors were independently associated with an increased incidence of psychiatric HCU, suggesting that persistent criminal behaviors in offender groups are indeed associated with psychiatric morbidity and HCU [24, 27, 31, 33].

This study had several limitations. As the DAABS cohort was selected by age, gender, and offense type, the information presented is not representative of a general prison population; it is rather a depiction of a small, selected group of violent male offenders with a history of imprisonment in young adulthood. Generalizing the current results to other offender groups or national settings other than Sweden or the Nordic countries should be preceded by careful deliberation. The psychiatric morbidity reported reflects diagnoses from specialist care; it likely underrepresents the actual prevalence of psychiatric disorders as not all cases may have received a diagnosis. The study also lacks information regarding primary care visits, and whether inpatient psychiatric HCU was voluntary or compulsory. This study had explorative aims and employed numerous statistical analyses. Consequently, risks of false positive findings were elevated and identified associations could thus dissipate in subsequent studies designed to test hypotheses further. The study’s strengths include a rigorous baseline measure, accomplished by experienced clinicians, using validated instruments in the DAABS total population study, followed longitudinally for a notable period through national registers and compared to a large general population group.

We suggest further research on interventions targeting violent offenders with psychiatric needs in transition from prison into the community [65]. As this study failed to replicate previous findings of an elevated total HCU in offenders [6, 31, 35], we propose additional reports exploring offenders’ interactions with healthcare, effects of psychiatric treatments, and associated societal costs.

In conclusion, we found an extensive and serious psychiatric morbidity in a cohort of violent offenders with an experience of imprisonment in young adulthood. Typically, the cohort interacted with psychiatric healthcare as inpatients rather than outpatients. Results indicated that in-depth knowledge of backgrounds, criminal behaviors, and psychiatric status help predict and may aid the planning of psychiatric services.

### Supplementary Information

Below is the link to the electronic supplementary material.

Supplementary file1 (PDF 514 KB)

## Data Availability

Researchers interested in accessing data that support the findings of this study should contact the corresponding author (andre.tarnhall@med.lu.se).
