# Correction to: Healthcare utilization and psychiatric morbidity in violent offenders: findings from a prospective cohort study

**DOI:** 10.1007/s00127-024-02629-x

**Published:** 2024-03-25

**Authors:** André Tärnhäll, Jonas Björk, Märta Wallinius, Peik Gustafsson, Eva Billstedt, Björn Hofvander

**Affiliations:** 1 Lund Clinical Research on Externalizing and Developmental Psychopathology, Department of Clinical Sciences Lund, Lund University, Lund, Sweden; 2 Department of Forensic Psychiatry, Region Skåne, Malmö, Sweden; 3 Centre of Ethics, Law and Mental Health, Department of Psychiatry and Neurochemistry, University of Gothenburg, Gothenburg, Sweden; 4 Department of Occupational and Environmental Medicine, Lund University, Lund, Sweden; 5 Research Department, Regional Forensic Psychiatric Clinic, Växjö, Sweden; 6 Gillberg Neuropsychiatry Centre, Institute of Neuroscience and Physiology, University of Gothenburg, Gothenburg, Sweden; 7 Department of Clinical Sciences Lund, Lund University, Lund, Sweden

**Correction to: Social Psychiatry and Psychiatric Epidemiology (2023) 58:617–628** 10.1007/s00127-022-02408-6

In this article, the data presented in Table 3 and Table 4 were incorrectly reported and have since been corrected. The corrected values are given in Table 3 and Table 4 below:

**Table 3:** Zero-inflated Poisson regression models using follow-up time to study the effect of trajectory groups and single risk factors from the DAABS baseline on total psychiatric health care visits in the DAABS cohort

	Effect size IRR (95% CI)	*p* value
Trajectory group		
Low-rate desisters	(reference group)	
Moderate-rate persisters	9.21 (5.12–16.59)	< 0.001*
High-rate late-peak persisters	8.41 (4.31–16.39)	< 0.001*
High-rate early-peak persisters	5.42 (2.79–10.51)	< 0.001*
High-rate inclining persisters	13.98 (7.84–24.91)	< 0.001*
Psychiatric background		
Mood disorders	2.43 (1.08–5.49)	0.03*
Anxiety disorders	2.77 (1.10–7.00)	0.03*
Psychotic disorders	1.28 (0.67–2.43)	0.45
Substance use disorders	3.07 (0.87–10.88)	0.08
Personality disorders	1.58 (0.87–2.87)	0.14
ADHD	1.75 (0.84–3.65)	0.14
Autism	1.51 (0.43–5.27)	0.52
Prior psychiatric care	4.30 (1.42–12.97)	0.01*
Socioeconomic background		
Low education	0.88 (0.44–1.77)	0.72
Never employed	1.87 (1.03–3.38)	0.04*
Born outside Sweden	1.04 (0.57–1.89)	0.90
Adversities and trauma		
Placement in a foster home	2.69 (1.57–4.61)	< 0.001*
Placement in institutional care	1.66 (0.91–3.05)	0.10
Being bullied	1.76 (1.01–3.08)	0.05*
Parental substance abuse	1.17 (0.68–2.02)	0.57
Parental violence, witness	1.04 (0.60–1.80)	0.89
Parental violence, victim	1.80 (1.06–3.07)	0.03*
Intellectual functioning		
General Ability Index	0.97 (0.95–0.99)	0.001*
Conduct problems		
Childhood-onset conduct disorder	1.24 (0.72–2.14)	0.41
Number of conduct disorder symptoms	1.03 (0.95–1.12)	0.45
Age at onset alcohol use	0.94 (0.83–1.07)	0.37
Age at onset substance use	0.97 (0.80–1.17)	0.72
Bullied others	0.94 (0.54–1.65)	0.84
Aggressive behaviors and psychopathic traits		
Life history of aggression score	1.01 (0.98–1.04)	0.50
Psychopathy checklist—revised score	1.04 (1.01–1.08)	0.02*

*p < 0.05

*CI* confidence interval, *DAABS* Development of Aggressive Antisocial Behaviour Study, *IRR* incidence rate ratio, *ADHD* attention deficit hyperactivity disorder

**Table 4:** Multivariable zero-inflated Poisson regression model using follow-up time to explain total psychiatric healthcare visits through risk factors and trajectory groups identified in the DAABS cohort

	Model 1 IRR (95% CI)	*p* value	Model 2 IRR (95% CI)	*p* value
Risk factors				
Anxiety disorders	2.36 (1.19–4.67)	.01*	2.08 (1.18–3.67)	.01*
Prior psychiatric care	2.07 (1.02–4.18)	.04*	1.61 (0.85–3.05)	.15
Placement in a foster home	2.60 (1.53–4.42)	< .001*	2.26 (1.36–3.75)	.002*
General Ability Index	0.97 (0.95–0.99)	.001*	0.97 (0.95–0.99)	.002*
Psychopathy Checklist—Revised score	1.06 (1.02–1.10)	.001*	1.06 (1.01–1.11)	.01*
Trajectory groups				
Low-rate desisters			(reference group)	
Moderate-rate persisters			4.02 (2.03–7.97)	< .001*
High-rate late-peak persisters			2.73 (1.33–5.63)	.007*
High-rate early-peak persisters			1.82 (0.79–4.18)	0.16
High-rate inclining persisters			3.60 (1.34–9.67)	0.01*

*p < .05

*CI* confidence interval, *DAABS* Development of Aggressive Antisocial Behaviour Study, *IRR* incidence rate ratio

The original article has been corrected.

