# Supplementary material for: Healthcare utilization and psychiatric morbidity in violent offenders: findings from a prospective cohort study
Source: Soc Psychiatry Psychiatr Epidemiol. 2022 Dec 27;58(4):617–28. doi: 10.1007/s00127-022-02408-6 (PMC10066109; doi:10.1007/s00127-022-02408-6)
Supplement: Supplementary file 1 — Supplementary file1 (PDF 514 KB) [file 127_2022_2408_MOESM1_ESM.pdf]

Healthcare Utilization and Psychiatric Morbidity in Violent Offenders: Findings from a Prospective Cohort Study

Supplementary Information

André Tärnhäll,[1, 2, 3] Jonas Björk,[4] Märta Wallinius,[1, 3, 5] Peik Gustafsson,[1] Eva Billstedt,[6] Björn Hofvander[1, 2, 3]

[1]Lund Clinical Research on Externalizing and Developmental Psychopathology, Department of Clinical Sciences Lund, Lund University, Lund, Sweden

[2]Department of Forensic Psychiatry, Region Skåne, Sweden

[3]Centre of Ethics, Law and Mental Health, Department of Psychiatry and Neurochemistry, University of Gothenburg, Gothenburg, Sweden

[4]Department of Occupational and Environmental Medicine, Lund University, Lund, Sweden

[5]Research Department, Regional Forensic Psychiatric Clinic, Växjö, Sweden

[6]Gillberg Neuropsychiatry Centre, Institute of Neuroscience and Physiology, University of Gothenburg, Sweden

Corresponding author: André Tärnhäll (andre.tarnhall@med.lu.se)

Table S1. Descriptive information of risk factors from the DAABS baseline measure

	N	***n* (%)/M (SD)**
**Psychiatric background**		
Mood disorders	264	141 (53)
Anxiety disorders	264	134 (51)
Psychotic disorders	265	20 (8)
Substance use disorders	266	224 (84)
Personality disorders	260	173 (66)
**Socioeconomic background**		
Low education	266	204 (77)
Never employed	266	44 (17)
Born outside Sweden	266	71 (27)
**Adversities and trauma**		
Placement in a foster home	266	68 (26)
Placement in institutional care	265	102 (38)
Being bullied	263	65 (25)
Parental substance abuse	263	108 (41)
Parental violence, witness	266	88 (33)
Parental violence, victim	265	107 (40)
**Intellectual functioning**		
General Ability Index	261	93.7 (10.9)
**Conduct problems**		
Childhood-onset conduct disorder	264	71 (27)
Number of conduct disorder symptoms	263	5.5 (3.4)
Age at onset alcohol use	246	14.1 (2.3)
Age at onset substance use	222	14.5 (2.5)
Bullied others	264	119 (45)
**Aggressive behaviors and psychopathic traits**		
Life History of Aggression score	264	30.1 (10.2)
Psychopathy Checklist – Revised score	259	17.7 (7.0)

ADHD: attention deficit hyperactivity disorder

Table S2. Categories of psychiatric diagnoses and prescribed drugs identified through ICD-10 codes and ATC codes, respectively

**Psychiatric morbidity**	**ICD-10 code(s)**
Major depressive disorders	F32-F39, F412
Anxiety disorders	F40-F42, F44-F48
Reaction to severe stress; adjustment disorders	F43
–Post-traumatic stress disorder	F43.1
Bipolar disorders	F30-F31
Psychotic disorders	F20-F29, F1x.5, F1x.7
–Primary psychotic disorders	F20-F29
–Substance-induced psychotic disorders	F1x.5, F1x.7
Alcohol use disorder	F10
Drug use disorders	F11-F19
Personality disorders	F60-F61
ADHD	F90
Autism	F84
Intellectual disability	F70F-F79
**Prescribed drugs**	**ATC code(s)**
Any psychotropic	N
Drug-classified pharmaceuticals	National definition
Analgesics	N02A, N02B
Antiepileptics	N03
Antipsychotics	N05A (excluding N05AN01)
Anxiolytics	N05B
Antidepressants	N06A
Mood stabilizers	N05AN01, N03AG01, N03AX09, N03AF01, N03AF02
Psychostimulants	N06B
Drugs, alcohol use disorder	N07BB
Drugs, opioid use disorder	N07BC

Table S3. Supplementary zero-inflated Poisson regression models using person-time to study the effect of single risk factors from the DAABS baseline on total psychiatric healthcare visits in the DAABS cohort

	**IRR (95% CI)**	***P* value**
**Psychiatric background**		
Major depressive disorders	1.27 (0.71–2.27)	.41
Bipolar disorders	2.00 (0.95–4.19)	.07
Alcohol use disorder1	.24 (0.71–2.17)	.45
Drug use disorders-	2.20 (0.99–4.88)	.05
–Cannabis	2.95 (1.39–6.27)	.005*
–Stimulants	2.19 (0.85–5.69)	.11
–Hallucinogens	1.58 (0.92–2.72)	.10
–Sedative-hypnotic-anxiolytic	2.64 (1.33–5.26)	.006*
–Heroin	1.38 (0.80–2.37)	.25
–Opioid analgesics	1.39 (0.81–2.39)	.24
–Other	1.71 (0.90–3.25)	.10
Extremely destructive substance abuse	1.10 (0.62–1.93)	75
Injection drugs	0.96 (0.48–1.93)	.91
Physical dependence	1.06 (0.58–1.92)	.85
Child and adolescent psychiatric healthcare utilization, any	2.48 (1.30–4.72)	.01*
-Child and adolescent psychiatric hospitalization	1.14 (0.53–2.46)	.74
Prior adult psychiatric healthcare utilization, any	1.78 (1.00–3.17)	.05
**Intellectual functioning**		
Verbal Comprehension Index	0.99 (0.97–1.02)	.49
Perceptual Organization Index	(0.96–0.98)	<.001*
**Conduct problems**		
Age at onset smoking	1.08 (0.92–1.25)	.35
Age at onset criminal behavior*	1.00 (0.93–1.09)	.93
**Aggressive behaviors and psychopathic traits**		
Physical aggression	1.02 (0.97–1.07)	.50
Consequences/antisocial behavior	1.02 (0.96–1.09)	.56
Self-directed aggression	1.06 (0.94–1.20)	.36
PCL – Factor 1	1.06 (0.98–1.14)	.12
PCL – Factor 2	1.09 (1.02–1.17)	.01*
PCL – facet 1	1.08 (0.90–1.30)	.41
PCL – facet 2	1.10 (0.97–1.24)	.13
PCL – facet 3	1.11 (0.98–1.26)	.10
PCL – facet 4	1.17 (1.06–1.30)	.002

Note: * p <.05 = significant
CI: confidence interval; DAABS: the Development of Aggressive Antisocial Behaviour Study; IRR: incidence rate ratio
